# Long-term Response to Nivolumab and Acute Renal Failure in a Patient with Metastatic Papillary Renal Cell Carcinoma and a PD-L1 Tumor Expression Increased with Sunitinib Therapy: A Case Report

**DOI:** 10.3389/fonc.2016.00250

**Published:** 2016-11-22

**Authors:** Juan Ruiz-Bañobre, Urbano Anido, Ihab Abdulkader, José Antúnez-López, Rafael López-López, Jorge García-González

**Affiliations:** ^1^Servizo de Oncoloxía Médica & Grupo de Oncoloxía Médica Traslacional, Instituto de Investigación Sanitaria de Santiago de Compostela (IDIS), Complexo Hospitalario Universitario de Santiago de Compostela, A Coruña, Spain; ^2^Servizo de Anatomía Patolóxica, Instituto de Investigación Sanitaria de Santiago de Compostela (IDIS), Complexo Hospitalario Universitario de Santiago de Compostela, A Coruña, Spain

**Keywords:** immunotherapy, long-term response, nivolumab, papillary renal cell carcinoma, programed death-ligand 1, renal cell carcinoma, sunitinib, vascular normalization

## Abstract

**Introduction:**

Papillary renal cell carcinoma (PRCC), which represents around 20% of renal cell carcinomas, is a heterogeneous disease that includes different tumor types with several clinical and molecular phenotypes. Nivolumab, a fully human IgG4 programed cell death protein 1 immune checkpoint inhibitor antibody, has shown not only an overall survival advantage when compared to everolimus but also a relatively good side-effect profile among patients with previously treated advanced or metastatic renal cell carcinoma.

**Case report:**

We describe a case of a young man diagnosed with PRCC that achieved a durable response to nivolumab despite a temporary suspension of the treatment due to a renal function side effect. To the best of our knowledge, it is the first renal failure secondary to nivolumab in a metastatic renal cell carcinoma patient.

**Concluding remarks:**

Nivolumab is a promising drug in patients with metastatic PRCC and long-term responses can be achieved. In case of acute renal failure secondary to this treatment, temporary therapy suspension and a low dose of systemic corticosteroids can recover renal function without a negative impact on treatment efficacy.

## Introduction

Over the last year, the treatment of advanced or metastatic renal cancer has been changed by the approval of several therapies. The results of a randomized, open-label, phase III study (CheckMate 025) have been recently published, in which nivolumab showed an overall survival advantage when compared to everolimus (25.0 vs. 19.6 months, respectively) with a relatively favorable side-effect profile (1). One of the inclusion criteria was the presence of any clear cell component in the tumor tissue specimen, excluding pure papillary renal cell carcinoma (PRCC), which accounts for around 20% of renal cell carcinomas. PRCC is a heterogeneous disease that includes different tumor types with different clinical and molecular phenotypes (2).

## Case Report

**Figure 1:**
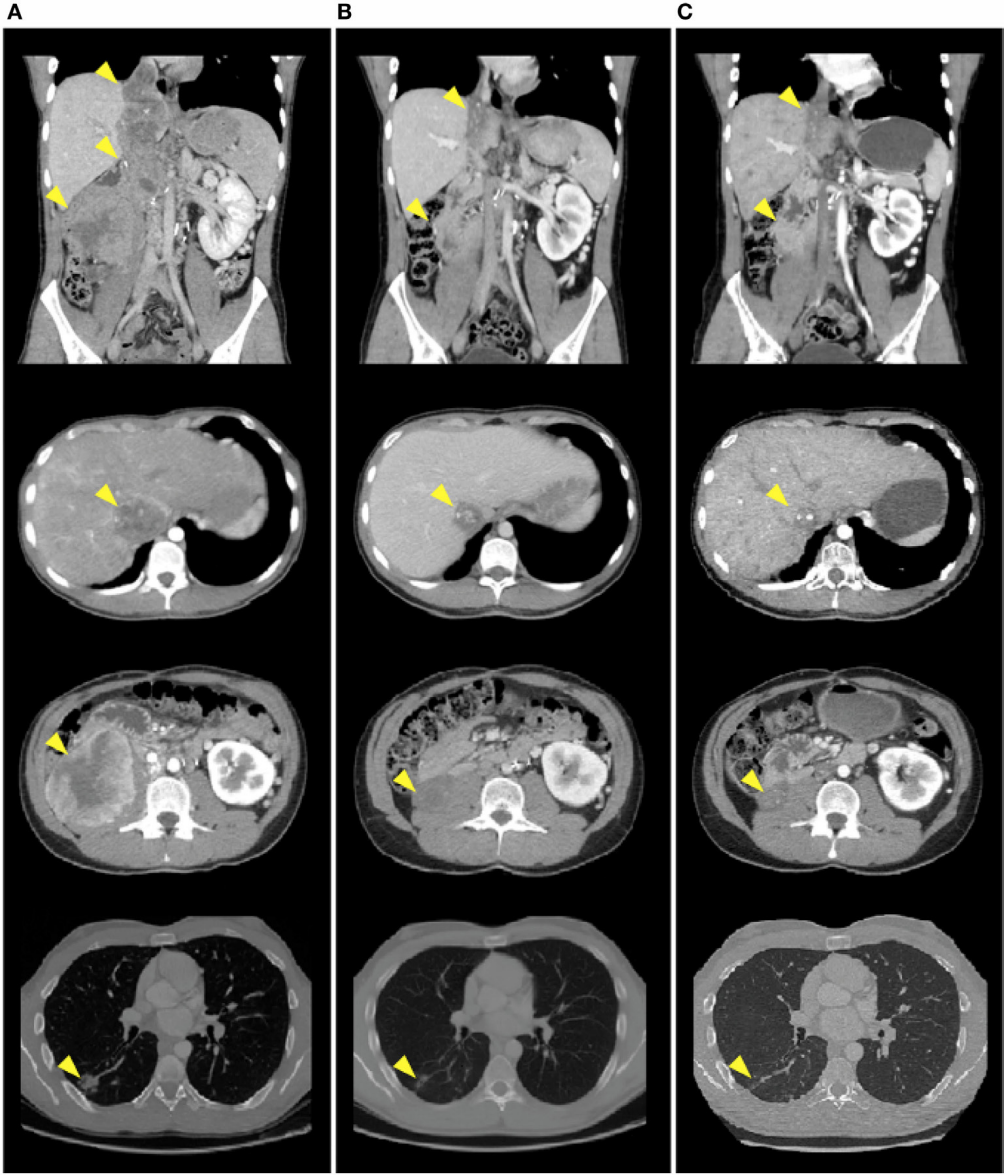
**CT scan images demonstrate a decrease in size of surgical site nodules, inferior vena cava tumor thrombus, paraaortic lymph node metastasis, and lung nodules after eight and fifteen infusions of nivolumab**. **(A)** Before nivolumab therapy initiation. **(B)** After eight infusions of nivolumab. **(C)** After fifteen infusions of nivolumab.

**Figure 2:**
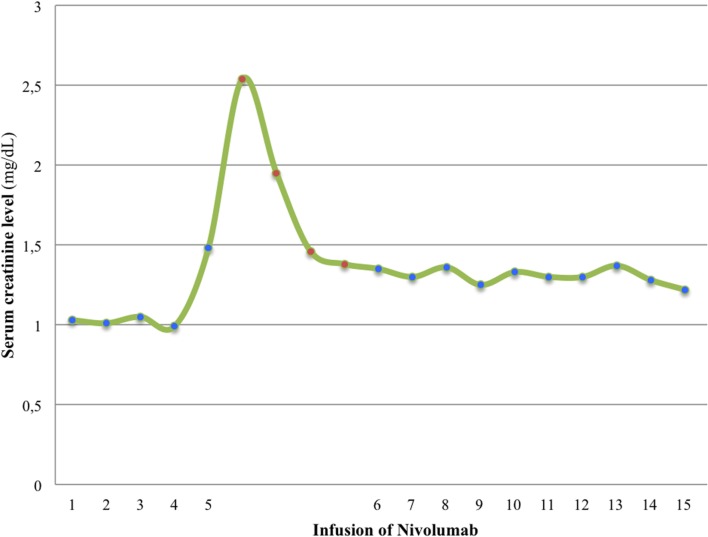
**Levels of serum creatinine rose 2 weeks after the fifth infusion of nivolumab**. Nivolumab therapy was discontinued and immediately prednisone treatment was initiated. Serum creatine levels diminished and nivolumab could be restarted.

A 20-year-old man was referred to our department diagnosed with recurrent PRCC after a nephrectomy for localized disease. He was previously healthy with no personal or family history of interest. Prior to resection of tumor recurrence, sunitinib was started on the 4/2 schedule (50 mg once/daily for 4 consecutive weeks on treatment followed by 2-week-off), continuing with the same treatment after surgery. Then, sunitinib was switched to the 2/1 schedule (50 mg once/daily for 2 consecutive weeks on treatment followed by 1-week-off) for toxicity, but in the end, the patient decided to suspend sunitinib due to side effects. Nine months after resection, he developed lung and paraaortic lymph node metastasis and the disease reappeared in the surgical site and the inferior vena cava tumor thrombus increased in size (Figure 1A). The patient started second-line treatment with nivolumab at 3 mg/kg every 2 weeks. Two weeks after the fifth infusion, a creatinine increment (2.5× above baseline level) and non-nephrotic range proteinuria were detected in blood and urine routine analyses, leading to an acute kidney injury grade 2 diagnosis. At that moment, after having ruled out obstruction and prerenal causes, we discontinued the nivolumab therapy and immediately started treatment with prednisone at 1 mg/kg every day and 3 days later, when the acute renal failure improved to grade 1, we initiated a progressive decrease of the corticosteroids dose (Figure 2). Nivolumab was restarted at the same dose 18 days after the suspension of prednisone, having been suspended for 39 days in total, with no worsening of the renal function. After eight infusions of nivolumab, a computed tomography (CT) scan showed a partial response at thoracic and abdominal levels (Figure 1B). At present, the patient continues with the treatment, there have been no new immune-related adverse events, and the last CT scan has shown a durable partial response (Figure 1C) after 15 infusions of treatment.

## Background and Discussion

Little is known about the efficacy of nivolumab in PRCC, and only one clinical case of a patient achieving a dramatic response has been reported in the literature (3). Here, we report, to our knowledge, the second case of a partial response to nivolumab achieved in a PRCC patient, with several outstanding features that make it noteworthy.

**Figure 3:**
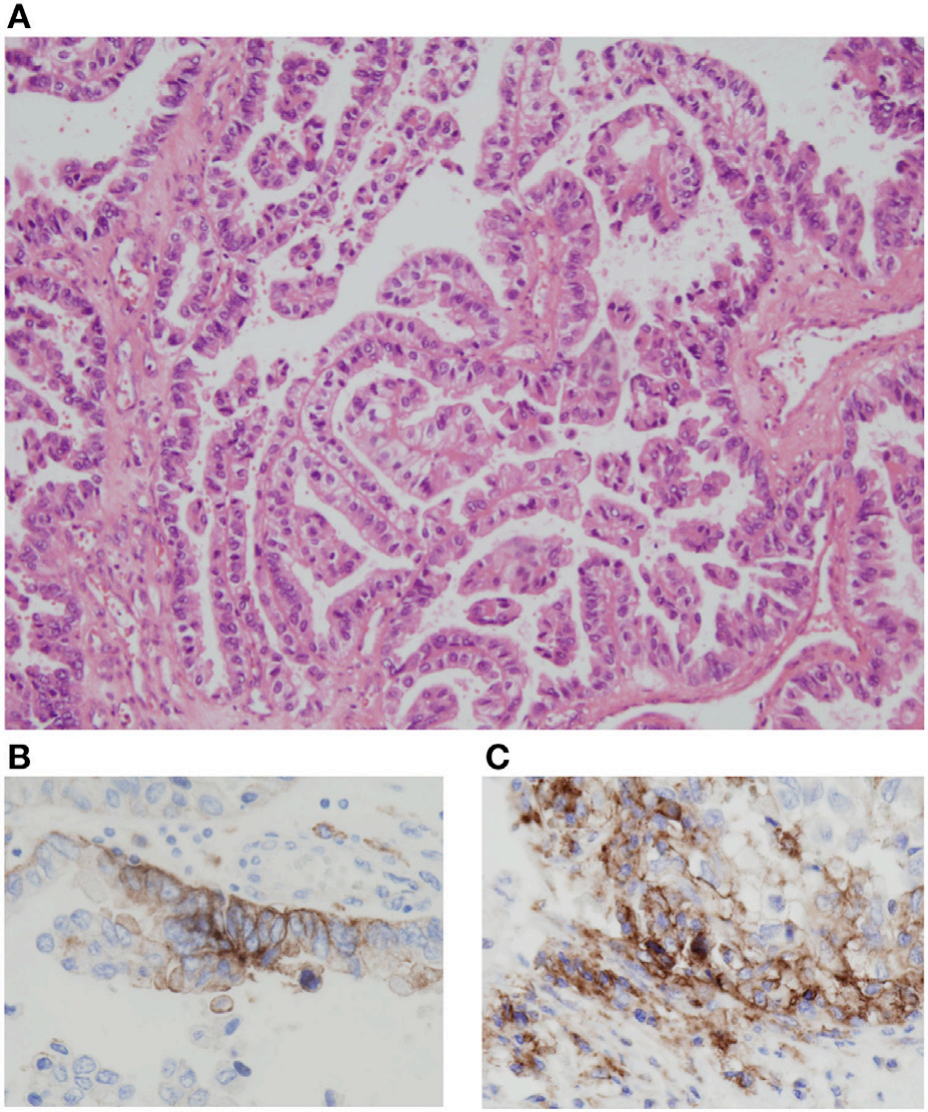
**Immunohistochemical analysis of PD-L1 expression was performed using PD-L1 IHC 22C3 pharmDx (qualitative immunohistochemical assay using monoclonal mouse anti-PD-L1, clone 22C3)**. Papillary renal cell carcinoma (hematoxylin and eosin stain; original magnification, ×10) **(A)**. Compared to the pre-sunitinib tumor tissue sample **(B)**, the number of stained tumor cells and stain intensity is higher in the post-sunitinib tumor tissue sample (original magnification, ×20) **(C)**. In the pre-treatment tissue sample, the PD-L1 expression is restricted to two foci as in the image **(B)**, while in the post-treatment tissue sample, there are multiple areas of focal expression. Quantitative PD-L1 expression: 15% **(B)** vs. 40% **(C)**. Two well-experienced pathologists (Ihab Abdulkader and José Antúnez-López) examined the immunohistochemical slides in a blinded fashion. Only tumor cells with a complete or partial circumferential linear plasma membrane staining at any intensity were considered for manual quantification.

It was recently reported that the expression of programed cell death-ligand 1 (PD-L1) in the immune component increased with pazopanib therapy, while CD8 expression decreased (4). It is unclear if the same happens with sunitinib (5, 6), but it may be an explanation to justify the response achieved in our case and in the case report by Geynisman. We analyzed PD-L1 tumor expression by immunohistochemistry and found that compared to the pre-sunitinib tumor tissue sample, the number of stained tumor cells and stain intensity is higher in the post-sunitinib tumor tissue sample (Table 1; Figure 3). CheckMate 025 clinical trial results do not support this hypothesis, because the expression of PD-L1 was not predictive of tumor response to nivolumab, but the immunohistochemical analysis in archival tissue samples obtained before previous regimens of antiangiogenic therapy probably distorts these results taking into account the dynamic and inducible nature of PD-L1 expression (7). On the other hand and related to this, there are different ongoing clinical trials to test combination therapies of anti-programed cell death protein 1 (PD-1) or anti-PD-L1 drugs with antiangiogenic drugs (8). The rationale for this approach is based on the fact that targeting the vascular endothelial growth factor (VEGF) axis may attenuate tumor-induced immunosuppression, allowing the tumor to become more responsive to immunotherapy. It is well known that the abnormal tumor vasculature can impede T effector cell infiltration into tumors and create a hypoxic and acidic tumor microenvironment that, among other actions, increases the accumulation of regulatory T (Treg) cells and myeloid-derived suppressor cells (MDSCs), decreases T cell priming, impairs T effector cells, and polarizes tumor-associated macrophages (TAMs) to the immune inhibitory M2-like phenotype. Moreover, hypoxia can also upregulate multiple immune-suppressive growth factors and cytokines. Pharmacologically induced “vascular normalization” can normalize the tumor vasculature and generate a more homogeneous distribution of perfused tumor vessels, facilitating the infiltration of T effector cells, while reducing MDSCs and Treg cells accumulation. In addition, mitigation of hypoxia and acidity by improved vascular perfusion polarizes TAMs to an immunostimulatory M1-like phenotype (9). Sunitinib, besides promoting these effects, blocks the signal transducer and activator of transcription 3 (STAT3) pathway, which is an immunosuppressive pathway favoring differentiation into regulatory cells and tumor growth. Decreasing STAT3 signaling diminishes the formation of Tregs cells and promotes the formation of effector T helper 1 (Th1) cells secreting interferon-γ (IFNγ) (10). In this context, as first described by Pardoll (11), the production of IFNγ by T cells upon recognition of their cognate antigen results in the reactive expression of PD-L1 by tumor cells and in the anergy induction in surrounding T cells (adaptive immune resistance).

**Table 1:** **Patient characteristics before nivolumab therapy**.

Age (years)	23
Gender	Male
ECOG PS	1
MSKCC group risk	Intermediate
Stage	IV
Tumor disease location	Surgical site nodules
	Paraaortic lymph node
	Lung nodules
	Inferior vena cava tumor thrombus
Tumor characteristics
Histological subtype	Papillary renal cell carcinoma
Fuhrman nuclear grade	3
PD-L1 expression (%)	
Pre-sunitinib sample	<5%
Post-sunitinib sample	20%
Previous therapies	Sunitinib
Previous surgeries	Nephrectomy
	Tumor recurrence resection

Long-term follow-up analysis of the patients involved in the phase I trial of nivolumab showed that 10 patients (29%) achieved objective responses with median response duration of 12.9 months (8.4–29.1+ months) (12). In the phase II trial of nivolumab, the objective response rate was 21.6% (95% CI 15.6–28.6), and median response duration was 23 months (80% CI 15.7–35.1) (13). In our case, the patient maintains a partial response after more than 8 months of starting treatment with nivolumab (response duration: 8.07+ months). Despite the fact that Geynisman et al. reported a case of a partial response in a patient with the same tumor subtype, it is the first case reported of a long-term response to nivolumab in a PRCC patient with well-defined survival data.

There are 14 cases clearly reported in the literature of renal side effects due to monotherapy with anti-PD-1 drugs, 7 of them in the metastatic melanoma setting, 6 in the non-small cell lung cancer setting, and 1 in the bladder carcinoma setting (14–17). Commonly, the clinical course is asymptomatic, with a gradual creatinine increment, and most patients improve with the use of low dose of corticosteroids. As an added complexity, our patient had a single kidney due to prior nephrectomy and, therefore, kidney biopsy was discarded then of evaluating the risks and benefits (18). Preliminary clinical data seem to show that systemic corticosteroids and other immunosuppressant drugs used for immune-related adverse events might not have such a negative impact on efficacy. A similar time to response and overall response rate was observed in melanoma patients treated with nivolumab who received systemic immunosuppressive therapy and those who did not (19, 20).

In an experimental animal model of autoimmune kidney disease, it is described that macrophage and T cells mediate injury and are prominent. Also, it is known that PD-L1 is constitutively expressed on kidney tubular epithelial cells and that intrarenal expression of PD-1 and its ligands increases in response to autoimmune-mediated renal inflammation. As demonstrated by Menke et al. (21) in animal models of autoimmune kidney disease, glomerular and tubular damage is greater in PD-L1 knockout mice compared to wild-type mice.

Taking this into account, we hypothesize that in humans the blockade of the PD-1/PD-L1 axis with nivolumab is responsible for the disinhibition of different immune cells related to renal damage, unbalancing the equilibrium toward a proinflammatory state, and, finally, facilitating the development of an acute renal impairment.

Brahmer et al. (22) investigated the PD-1 occupancy on circulating T cells over time using flow cytometric analysis. PD-1 occupancy after one infusion of nivolumab appeared to be dose independent, with a mean plateau occupancy of 72% at ≥57 days. These data could explain why the effectiveness of treatment does not appear to have been compromised in the case of our patient despite discontinuation of the treatment for 39 days due to acute renal failure grade 2.

## Concluding Remarks

Nivolumab is a promising drug in patients with metastatic PRCC, and long-term responses can be achieved. Although few cases have been described, the appearance of immune-related adverse events can be a problem to solve during anti-PD-1 therapies. To improve its management and to clarify the peculiarities in the pathogenesis of acute renal failure induced by anti-PD-1 drugs, further clinical and translational trials must be developed. In case of acute renal failure secondary to this treatment, temporary therapy suspension and a low dose of systemic corticosteroids can recover renal function without a negative impact on treatment efficacy.

## Ethics Statement

Due to the observational nature of this case report, no formal ethics approval was required. The patient agreed and provided written informed consent. Written informed consent was obtained from the patient for publication of this case report and any accompanying images.

## Author Contributions

Study concept and design: JR-B. Acquisition, analysis, and interpretation of data: JR-B, JG-G, RL-L, JA-L, IA, and UA. Drafting of the manuscript: JR-B. Critical revision of the manuscript for important intellectual content: JG-G, RL-L, JA-L, IA, and UA. All the authors read and approved the final manuscript. All the authors agree to be accountable for all aspects of the work in ensuring that questions related to the accuracy or integrity of any part of the work are appropriately investigated and resolved.

## Conflict of Interest Statement

The authors declare that the research was conducted in the absence of any commercial or financial relationships that could be construed as a potential conflict of interest.

## Abbreviations

CT, computed tomography; IFNγ, interferon-γ; MDSCs, myeloid-derived suppressor cells; PD-1, programed cell death protein 1; PD-L1, programed cell death ligand 1; PRCC, papillary renal cell carcinoma; STAT3, signal transducer and activator of transcription 3; TAMs, tumor-associated macrophages; Th1, T helper 1; Treg, regulatory T; VEGF, vascular endothelial growth factor.
